# Role of PCSK9 inhibitors in venous thromboembolism: current evidence and unmet clinical needs

**DOI:** 10.1093/ehjcvp/pvae076

**Published:** 2024-10-15

**Authors:** Marco Zuin, Alberto Corsini, Chiara Dalla Valle, Catia De Rosa, Alessandro Maloberti, Marco Mojoli, Massimiliano Rizzo, Francesco Ciccirillo, Alfredo Madrid, Carmine Riccio, Massimo Grimaldi, Furio Colivicchi, Fabrizio Oliva, Pier Luigi Temporelli, Associazione Nazionale Medici Cardiologi Ospedalieri (ANMCO) Working Group on Cardiological Chronicity

**Affiliations:** Department of Translational Medicine, University of Ferrara, Via Luigi Borsari 46, Ferrara 44121, Italy; Department of Cardio-Thoraco-Vascular Sciences and Public Health, University of Padova, via Giustiniani 2, Padova 35128, Italy; Department of Pharmacological and Biomolecular Sciences, University of Milan, Milan 20133, Italy; Department of Cardiology, West Vicenza General Hospital, Arzignano 36071, Italy; Department of Cardiology, Ospedale Mauriziano Umberto I, Torino 10128, Italy; Cardiac Rehabilitation Unit, Cardiology 4, ASST Grande Ospedale Metropolitano Niguarda, Milano 20162, Italy; Division of Cardiology, Ospedale Santa Maria degli Angeli, Azienda Ospedaliera Friuli Occidentale (ASFO), Pordenone 33170, Italy; Department of Cardiology, Policlinico Umberto I, Roma 00161, Italy; Department of Cardiology, Ospedale Vito Fazzi, Lecce 73100, Italy; Department of Cardiology, AORN Cardarelli, Napoli 80131, Italy; Cardiovascular Department, Sant'Anna e San Sebastiano Hospital, Caserta 81100, Italy; Department of Cardiology, Ospedale Generale Regionale “F. Miulli”, Acquaviva delle Fonti 70021, Italy; Clinical and Rehabilitation Cardiology Unit, San Filippo Neri Hospital, Roma 00135, Italy; Cardiology Unit, ASST Grande Ospedale Metropolitano Niguarda, Milano 36071, Italy; President, Associazione Nazionale Medici Cardiologi Ospedalieri (ANMCO), Florence 50121, Italy; Division of Cardiac Rehabilitation, Istituti Clinici Scientifici Maugeri, IRCCS, Gattico-Veruno 28013, Italy

**Keywords:** Venous Thromboembolism thromboembolism, PCSK9 inhibitors, Prevention

## Abstract

Proprotein convertase subtilisin/kexin type 9 inhibitors (PCSK9i) have recently emerged as promising therapeutic agents for lowering low-density lipoprotein cholesterol and reducing the risk of cardiovascular events. Moreover, preliminary evidence from randomized controlled trials (RCTs) suggests that PCSK9i may also offer beneficial effects for patients following venous thromboembolism (VTE), with the most significant reductions in risk appearing over time, particularly beyond the first year of treatment. However, there is a lack of randomized controlled data supporting their efficacy and safety in conjunction with standard anticoagulation therapy. This article aims to critically evaluate the existing evidence for the use of PCSK9i as a complementary therapy for VTE risk reduction, while also identifying unmet clinical and research needs and proposing potential strategies to address these knowledge gaps.

## Introduction

Venous thromboembolism (VTE), encompassing deep vein thrombosis (DVT) and acute pulmonary embolism, remains the third leading cause of cardiovascular mortality in western countries after myocardial infarction and stroke.^1^ Recent investigations have challenged the notion that VTE and atherosclerosis are distinct clinical entities, revealing shared risk factors between them.^2–4^ Despite this understanding, preventive therapies for VTE, beyond standard anticoagulation, are currently lacking.

Anticoagulation therapy is essential for managing VTE, but it has several limitations.^5^ Anticoagulants are associated with a variable risk of clinically significant bleeding and do not reduce the incidence of post-thrombotic syndrome.^6,7^ Additionally, direct oral anticoagulants lack sufficient head-to-head trials comparing their efficacy and safety for long-term secondary prevention of VTE. ^7^ The optimal duration of anticoagulation remains uncertain, with current guidelines recommending 3–6 months for provoked VTE and indefinite therapy for unprovoked VTE in patients with a low-bleeding risk.^8^ These limitations underscore the need for improved anticoagulant strategies and adjunct therapies to enhance VTE management without increasing bleeding risks, particularly for frail and comorbid patients. Over the last two decades, evidence from observational and intervention studies have suggested a beneficial effect of statin use on the risk of VTE, either in primary or secondary prevention.^9–12^ More recently, proprotein convertase subtilisin/kexin type 9 inhibitors (PCSK9i) have emerged as innovative promising therapeutic agents in the management of cardiovascular diseases (CVDs), especially in reducing the risk of atherosclerotic events.^13^ While their primary mechanism of action involves lowering low-density lipoprotein cholesterol (LDL-C) levels through inhibition of PCSK9-mediated degradation of LDL receptors, recent research suggests that PCSK9i may also play a role in reducing the risk of VTE.^14^ However, data supporting the effectiveness and safety of PCSK9i in reducing VTE prevention, especially compared to anticoagulants, are lacking.

The aim of the present manuscript is to review existing evidence regarding the risk reduction for VTE associated with PCSK9i, highlighting unaddressed clinical and research needs and suggesting potential strategies to resolve these knowledge gaps.

## Venous thromboembolism and atherosclerosis

Several lines of evidence have confirmed a link between arterial and venous thrombosis, especially in patients with idiopathic VTE.^15–17^ Hyperlipidemic patients exhibit increased thrombin generation and higher platelet activation rates, creating a prothrombotic state and promoting endothelial venous deterioration.^18^ Furthermore, lipoprotein(a) (Lp(a)), which is structurally and functionally similar to plasminogen, may compete with plasminogen for fibrin binding sites, thereby impairing fibrinolysis.^19,20^ Moreover, both clinical and preclinical atherosclerosis are more common in patients with spontaneous VTE compared to healthy individuals and the incidence of fatal or non-fatal symptomatic atherosclerotic disease has been reported to be nearly twice as high in patients with VTE.^2,21,22^ These associations have been confirmed by various observational and randomized controlled trials, suggesting that statins may reduce the risk of VTE due to their pleiotropic effects, beyond merely lowering cholesterol levels.^10–12^ However, whether non-statin lipid-lowering agent, such as PCSK9i, may reduce the risk of VTE events, remains matter of debate.^23,24^

## PCKS9i and risk of VTE: evidence from RCTs

The main biological function of PCSK9i is the degradation of the LDL receptor (LDLR) in hepatocytes leading to lower LD-C uptake by the liver.^25,26^ PCSK9 is a serine protease synthesized in the cell as a soluble zymogen and converted into its active form after an autocatalytic process in the endoplasmic reticulum.^25^ This protease represents a critical regulator of cholesterol homeostasis as it acts as a counter regulator of LDLR expression on the cell surface.^26^ Specifically, PCSK9 is mainly secreted by hepatocytes, but is also expressed in the arterial wall, where it can influence local haemostasis and directly contribute to the development of atherosclerosis.^26^ Given the strong correlation between dyslipidaemia and CVD, reducing LDL-c through LDLR-PCSK9 axis inhibition drastically reduces the risk of CVD. Over the last decade, several PCSK9 inhibitors (PCSK9i) have been developed, including two human monoclonal antibodies, alirocumab and evolocumab.^27,28^ Specifically, from a pharmacological perspective, PCSK9i bind circulating PCSK9, preventing the formation of the PCSK9-LDL-LDLR complex and subsequent LDLR turnover.^29^

**Figure 1:**
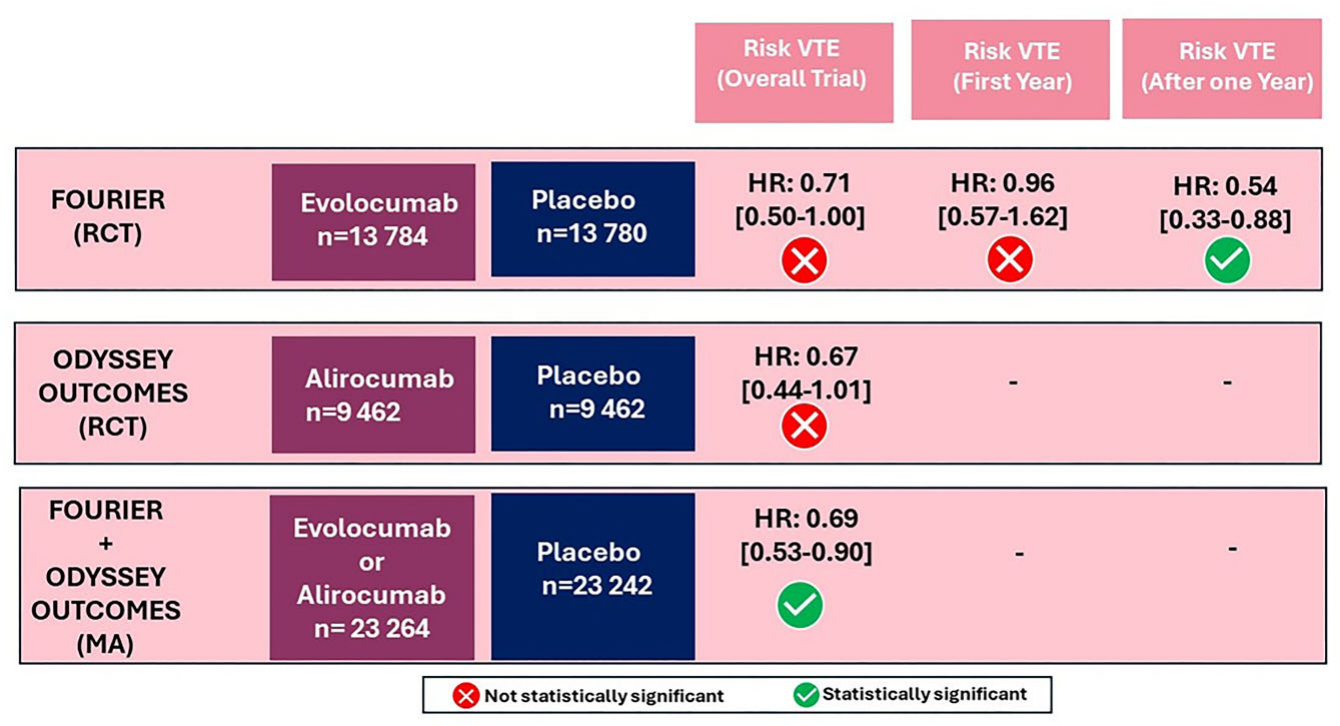
Main results of randomized controlled trials and meta-analysis evaluating the effect of PCSK9i on the risk of venous thromboembolism. RCT, randomized controlled trial; MA, meta-analysis; HR, Hazard ratio; [], 95% confidence interval.

Few RCTs have investigated the association between PCKS9i and the risk of VTE. A recent meta-analysis combining data from the FOURIER (NCT01764633, evolocumab, *n* = 27 564) and the ODYSSEY OUTCOMES (NCT01663402, alirocumab, *n* = 18 924) trials revealed that patients receiving PCSK9i experienced a 31% reduction in the risk of VTE compared to those on placebo [Hazard ratio (HR) = 0.69; 95% CI 0.53–0.90] (*Figure 1*).^30^ Conversely, a subsequent analysis based on the FOURIER trial, showed that PCSK9 inhibition did not influence VTE occurrence within the first years of treatments (HR 0.96; 95% CI 0.57–1.62). However, after that period, the use of a PCSK9i significantly reduced the risk of VTE of about 46% (HR 0.54; 95% CI 0.33–0.88).^30^ Furthermore, VTE reduction resulted not associated with LDL-C lowering but rather with baseline levels of Lp(a) and degree of lowering. Specifically, in patients with elevated baseline Lp(a) levels, evolocumab led to a reduction in Lp(a) by 33 nmol/L and a corresponding 48% decrease in VTE risk (HR 0.52; 95% CI: 0.30–0.89). However, in individuals with lower baseline Lp(a) levels, evolocumab led to a modest reduction of only 7 nmol/L in Lp(a) and no impact on VTE risk (p_interaction_ for HR 0.087 and p_heterogeneity_ for absolute risk reduction—ARR—0.037).^30^ Overall, these results suggest that VTE reduction is a class effect of PCSK9i probably reducing the VTE risk by decreasing Lp(a) levels.^30^ Nevertheless, however, results of genetic studies do not support a causal effect of Lp(a) levels on the risk of VTE.^31,32^ The underlying pathophysiological mechanism appears to stem from the structural resemblance between Lp(a) and plasminogen, potentially hindering fibrin binding, impairing tissue plasminogen activator function, and disrupting fibrinolysis, thereby promoting a hypercoagulable state.^33,34^ These results have been recently confirmed by a large network meta-analysis, which demonstrated that the greatest reduction in VTE was achieved in patients treated with a combination of PCSK9 inhibitors and high-intensity statins, compared to a placebo (relative risk—RR—: 0.59, 95% CI: 0.43–0.80).^35^

## Pathophysiological aspects

Various *in vitro* and *in vivo* analyses have indicated that alterations in PCSK9 may influence blood coagulation. Wang *et al.* demonstrated that PCSK9-deficient (PCSK 9−/−) mice developed significantly smaller venous thrombi compared to wild-type mice following inferior vena cava ligation.^36^ Additionally, levels of soluble P-selectin (sP-selectin), a biomarker for platelet and endothelial activation, were notably lower in PCSK9-deficient mice than in controls after thrombosis induction, suggesting impaired platelet function and reduced endothelial activation.^36^ Notably, humans with unprovoked DVT and endothelial dysfunction also exhibited increased circulating sP-selectin levels.^37,38^ Elevated LDL-C levels have been associated with heightened oxidative stress, leading to the generation of oxidized-LDL (ox-LDL), which plays a crucial role in inflammation-driven thrombosis by activating platelet receptors CD36 and lectin-like oxidized low-density lipoprotein receptor 1(LOX-1).^38–40^ Moreover, by lowering plasma LDL-C levels, PCSK9i potentially deplete cholesterol in platelet membranes, reducing their reactivity and pro-coagulant activity.^41,42^ Likewise, the attenuation of platelet activation by PCSK9i may mitigate ox-LDL generation, disrupting the harmful cycle perpetuating platelet activation. PCSK9i also increase HDL levels, which can directly inhibit platelet aggregation. In addition, several experimental and clinical studies have also demonstrated the pro-thrombotic effect of Lp(a). PCSK9i also have the additional benefit of reducing Lp(a) concentration by 20–25%.^43^ In line with this, a meta-analysis involving approximately 14 000 patients confirmed that elevated levels of Lp(a) are significantly associated with an increased incidence of VTE (Odds ratio—OR—: 1.56, 95% CI: 1.36–1.79).^44^

**Figure 2:**
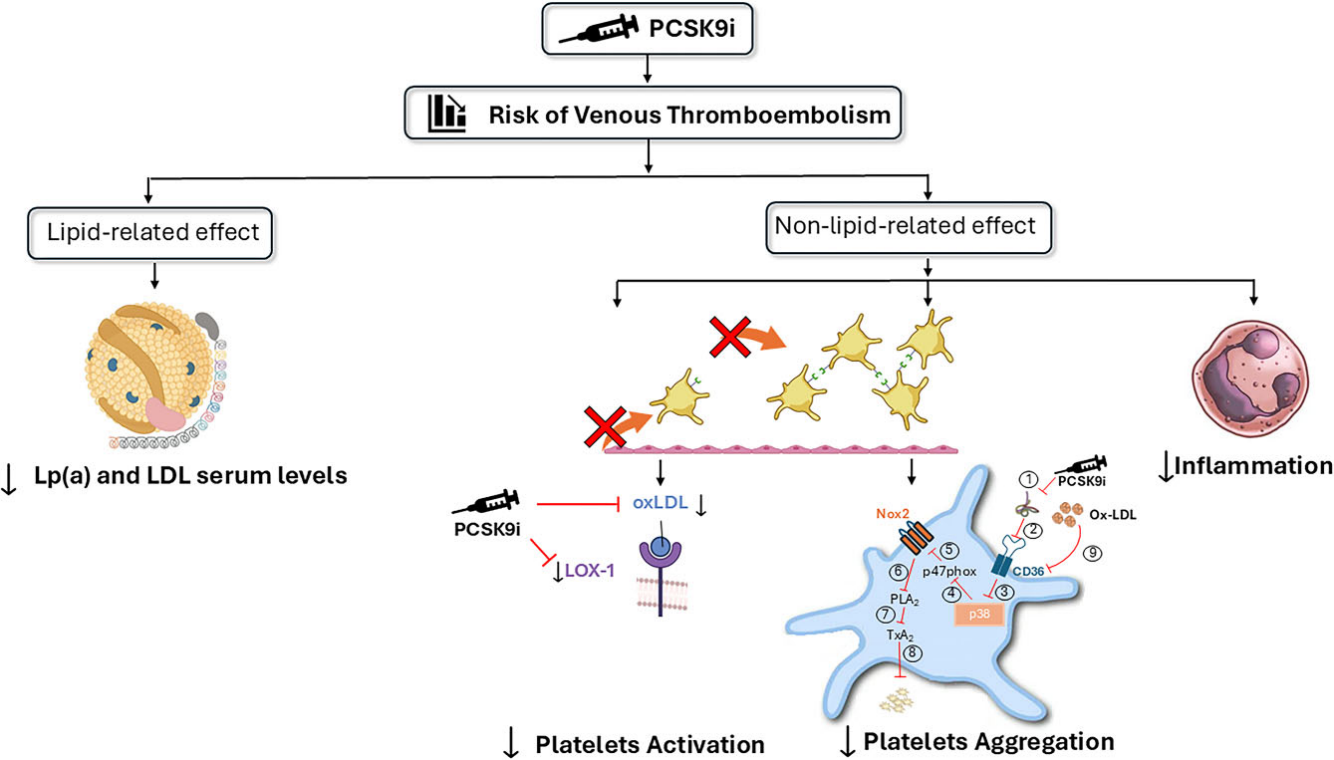
PCSK9i can reduce the risk of VTE through both lipid-mediated and non-lipid-mediated effects. Specifically, PCSK9 inhibition decreases platelet activation by reducing platelet oxidized LDL receptor (LOX-1) expression and the generation of oxidized LDL (ox-LDL). PCSK9i significantly reduce circulating PCSK9 levels (1),^48^ inhibiting its interaction with the CD36 receptor (2) and thereby decreasing the phosphorylation of p38MAPK (3) and the translocation of p47phox from the cytosol to the membrane (4), which is required for Nox2 activation (5). The lack of p47 phosphorylation reduces the activation of Phospholipase A_2_ (PLA_2_) (6), which is responsible for the release of arachidonic acid and thromboxane A2 (TxA_2_) (7), ultimately decreasing platelet aggregation (8). Furthermore, the reduction in oxidized LDL prevents the amplification of CD36 signalling and subsequent platelet activation (9).^49^

Additionally, PCSK9 can impact coagulation through its effect on clotting factor VIII (FVIII).^45^ The LDL receptor-related protein 1 (LRP-1) mediates the endocytosis and degradation of FVIII, suggesting a potential mechanism through which PCSK9 may contribute to thrombus formation.^46^ Specifically, PCSK9 increases FVIII levels by reducing LRP-1 expression.^46^ However, PCSK9 inhibitors have been shown to reduce FVIII levels by enhancing LRP-1 expression in mouse models.^47^ Hence, PCSK9i enhance the LDL receptor on the hepatocyte surface, promoting the clearance of factor VIII and tissue factor, thereby lowering the risk of VTE. Moreover, current evidence indicates that PCSK9i may reduce the risk of VTE through both lipid- and non-lipid-related effects, including the reduction of Lp(a) serum levels and the inhibition of platelet aggregation and activation (*Figure 2*).^48^  ^49^ Nonetheless, further investigations are necessary to gain a deeper understanding of these underlying pathophysiological mechanisms.

## Current unmet needs and potential solutions

Despite the current growing of evidence supporting the use of PCSK9i for the prevention of atherosclerotic CVD, additional RCTs are necessary to identify those patients who would benefit from their administration for preventing VTE, either in primary or secondary prevention.^50^ Unfortunately, no specific RCTs addressing this issue are currently underway, widening the current knowledge gap. Hopefully, new studies, also investigating the potential role of inclisiran, a small interfering RNA agent used to lower LDL-C, will clarify the potential role of PCSK9i in patients with dyslipidaemia.

Targeting platelet function and inflammation is crucial for reducing the risk VTE as well as the risk of atherothrombotic events.^51–54^ Growing evidence indicates that platelets play a significant role in both the initiation and propagation of venous thrombi.^55^ Elevated markers of platelet activation, such as soluble P-selectin, have been observed in patients with acute VTE and those at high risk, suggesting that platelets may serve as valuable biomarkers for VTE.^56,57^ Furthermore, antiplatelet therapy, such as aspirin, has demonstrated effectiveness in reducing the risk of VTE after orthopaedic surgery and in preventing recurrent VTE.^58^ Additionally, the presence of large, reactive platelets is associated with an increased risk of both DVT and myocardial infarction, highlighting the shared mechanisms underlying arterial and venous thrombosis.^59^ While the mechanisms of platelet activation may differ between arterial and venous thrombosis, the final pathway of platelet aggregation and thrombus formation is common to both conditions. Factors that enhance platelet reactivity, particularly inflammation, can predispose individuals to both VTE and ACS. Therefore, addressing platelet function and the inflammatory response not only targets the thrombotic process but also mitigates the risk of recurrent events.^60^ By focusing on these pathways, we can develop more effective therapeutic strategies that improve outcomes in patients at risk for both VTE and ACS, ultimately enhancing patient care and reducing the burden of thromboembolic diseases.

**Figure 3:**
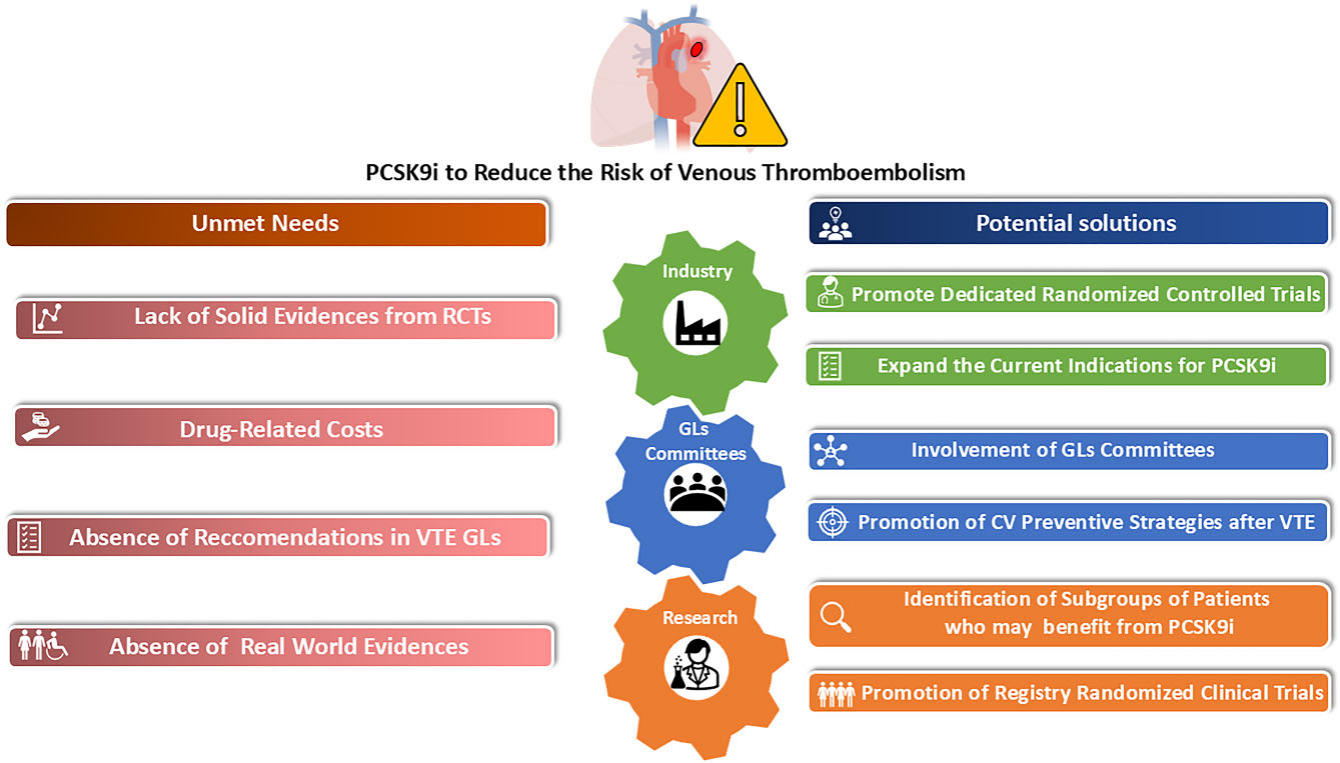
Current unmet needs and potential solutions for promoting the use of PCSK9 inhibitors as an adjunctive strategy to reduce the risk of venous thromboembolism. GLs, guidelines; VTE, venous thromboembolism.

However, despite preliminary evidence suggests that PCSK9i may reduce VTE risk through lipid and non-lipid effects, the underlying mechanisms remain incompletely understood (*Figure 3*). Several factors contribute to this lack of interest. Firstly, existing data from RCTs have demonstrated a significant relative risk reduction for VTE but only a modest decrease in the absolute event rate. Secondly, from a practical point of view, implementing a universal approach of administering PCSK9i to mitigate VTE risk may not be financially sustainable for the larger part of national healthcare systems. Additionally, data assessing the cost-effectiveness of PCSK9i in the VTE prevention are lacking. Finally, current international guidelines on VTE management lack specific recommendations for lifestyle changes, alternative pharmacological preventive strategies not based on standard anticoagulation following the acute event. Although the evidence for the use of PCSK9i for the prevention and reduction of atherosclerotic cardiovascular events continues to increase,^61^ VTE appears to remain a secondary concern compared to myocardial infarction and ischemic stroke. Probably, in some selected comorbid VTE patients, the administration of PCSK9i, as an adjunctive preventive treatment may be a valid strategy for preventing new CVD in the long term.

The identification of these patients remains a priority and an important unmet need. These unmet needs may be resolved by conducting specific RCTs and involving international guidelines committees working on both VTE and dyslipidaemia. Despite RCTs are generally considered as the gold standard for studying causal relationships as randomization eliminates much of the bias inherent with other study designs,^62^ performing them remains often challenging due to funding limitations, complex regulatory requirements, and infrastructure needs. Moreover, RCTs are often not fully representative of the real-world population. To address these challenges, alternative data sources with rigorous bias-minimization techniques may be pursued. These include propensity score matching, independent blinded outcome adjudication, and central core-lab analyses. Additionally, registry-based randomized controlled trials, also known as pragmatic trials, offer a promising alternative for conducting robust analyses. These trials, characterized by lower costs, enhanced generalizability, rapid enrolment, and facilitated follow-up, may provide an innovative approach for evaluating the effectiveness and safety of PCSK9i in real-world settings, mitigating the inherent biases of observational studies.

## Conclusions

In conclusion, compelling evidence supports the reduction of VTE risk with PCSK9i, including evolocumab and alirocumab alone or in combination with high-intensity statin. Such decrease seems to correlate with the extent of Lp(a) reduction rather than LDL cholesterol lowering, underscoring the potential importance of Lp(a) as a mediator of systemic thromboembolism. PCKSK9 pharmacologic inhibition may reduce the risk of VTE events, besides lipid lowering. Considering that VTE is a potentially preventable disease, identifying effective primary and secondary preventive strategies that also reduce overall cardiovascular risk represents a promising approach. However, further studies are needed to confirm available preliminary results. Important complementary data, elucidating the role of Lp(a) will be obtained from some ongoing phase 2 and phase 3 trials investigating RNA therapeutics targeting Lp(a).

## Data Availability

No new data were generated or analysed in support of this research.
